# Supplementary material for: A unique sigma/anti-sigma system in the actinomycete Actinoplanes missouriensis
Source: Nat Commun. 2023 Dec 20;14:8483. doi: 10.1038/s41467-023-44291-y (PMC10733313; doi:10.1038/s41467-023-44291-y)
Supplement: Supplementary file 1 — Supplementary Information [file 41467_2023_44291_MOESM1_ESM.pdf]

Supplementary Information

A unique sigma/anti-sigma system in the actinomycete *Actinoplanes missouriensis*

Takeaki Tezuka,[a,b,#,*] Kyota Mitsuyama,[a,#] Risa Date,[a,#] Yasuo Ohnishi[a,c,*]

[a]Department of Biotechnology, Graduate School of Agricultural and Life Sciences, The University of Tokyo, Bunkyo-ku, Tokyo, Japan

[b]Graduate School of Infection Control Science, Kitasato University, Kitasato University, Minato-ku, Tokyo, Japan

[c]Collaborative Research Institute for Innovative Microbiology, The University of Tokyo, Bunkyo-ku, Tokyo, Japan

[#]These authors equally contributed to this work.

*Address correspondence to Takeaki Tezuka, atezuka@mail.ecc.u-tokyo.ac.jp; Yasuo Ohnishi, ayasuo@mail.ecc.u-tokyo.ac.jp

Contents

Fig. S1. Heat resistance of sporangiospores and zoospores.

Fig. S2. Numbers of spores released from sporangia.

Fig. S3. SEM observation of mycelia and sporangia.

Fig. S4. Observation of sporangium dehiscence using phase-contrast microscopy.

Fig. S5. Number of spores released from the sporangia after prolonged incubation.

Fig. S6. BACTH assays for SipA and σ^{SsdA}.

Fig. S7. AlphaFold- or AlphaFold-Multimer-based prediction of the SipA or SipA-σ^{SsdA} complex structures.

Fig. S8. Transcript levels of *sipA*, *ssdA* and 17 genes under the direct control of σ^{SsdA}-recognizing promoters.

Fig. S9. Nucleotide sequences of 17 σ^{SsdA}-recognizing promoter-containing regions.

Fig. S10. Schematic representation of the *rsdR*-*rsdK* operon and domains of RsdK and RsdR and transcript levels of *rsdK* and *rsdR*.

Fig. S11. Observation of sporangia and zoospores using phase-contrast microscopy.

Fig. S12. Distribution of *sipA* and *ssdA* homologues in actinomycetes.

Table S1. Mutations identified in the sporangium dehiscence-deficient mutant strains.

Table S2. Mutations identified in the sporangium dehiscence-competent suppressor strains.

Table S3. Primers used in this study.

Supplementary Note 1

Supplementary Note 2

Supplementary References

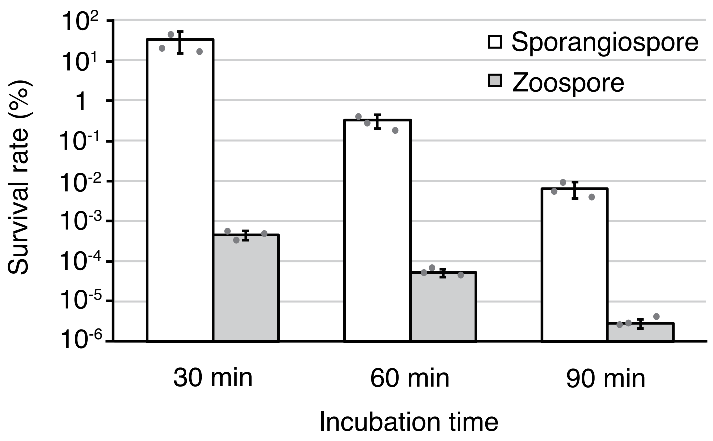

Fig. S1. Heat resistance of sporangiospores and zoospores. Sporangia harvested from mycelia were suspended into distilled water and the suspension was divided into two equal parts. Sporangium dehiscence was induced by adding the dehiscence-inducing solution to one part, while the other part did not undergo this procedure. Suspensions of sporangia or zoospores were incubated at 50°C for 90 min. Aliquots of the suspensions were sampled before and at 30, 60, and 90 min after the onset of incubation. A portion of the sample was cultivated on YBNM agar at 30°C for 2 days, and the number of growing colonies was counted to estimate the colony-forming unit (CFU) value of the sample. The heat resistance of sporangiospores and zoospores is shown as the survival rate by taking the CFU before incubation at 50°C as 100% in each suspension. A sporangium contains 100-200 spores, and therefore forms a much larger colony than a zoospore. Because only large colonies were observed even after heat treatment, a large portion of sporangiospores in the sporangium seemed to be alive when the sporangium survived heat treatment. The values represent mean ± standard error of three biological replicates. Source data are provided as a Source Data file.

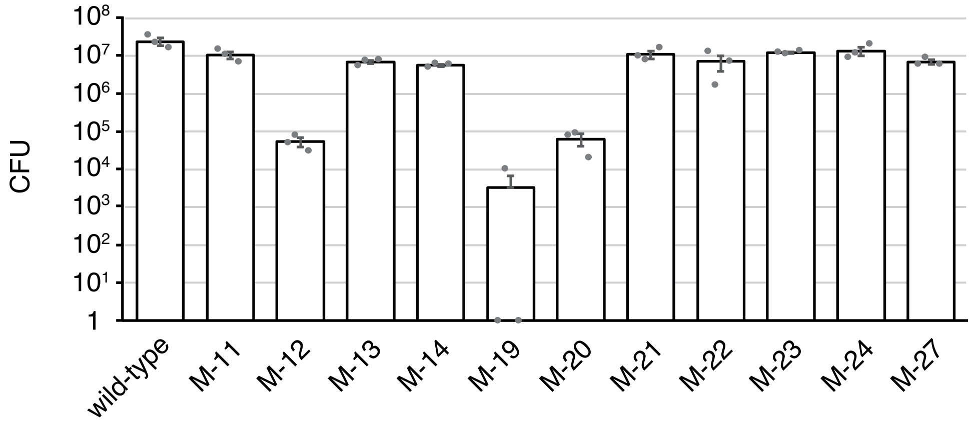

Fig. S2. Numbers of spores released from sporangia. The wild-type and 11 mutant strains were cultivated on HAT agar at 30°C for 7 days. Zoospores released from the sporangia formed on one HAT agar plate by pouring 25 mM NH_4HCO_3 solution were counted as CFU on YBNM agar. Data are the means of three biological replicates ± standard error. Source data are provided as a Source Data file.

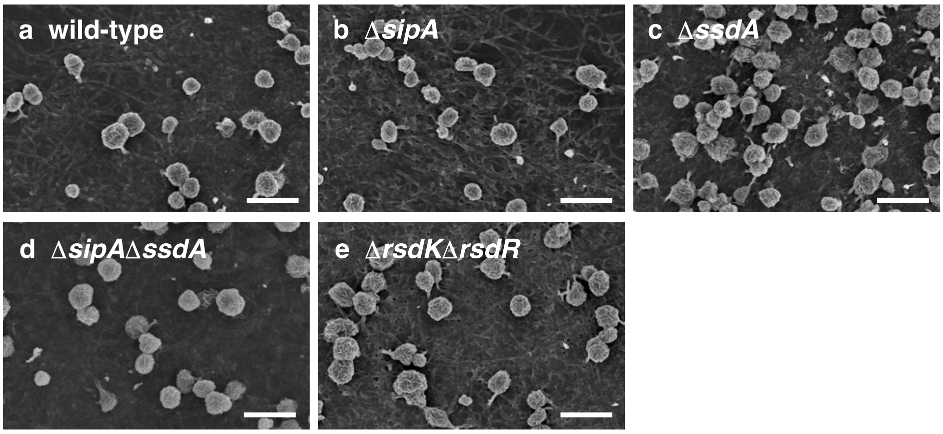

Fig. S3. SEM observation of mycelia and sporangia. Each strain was cultivated on HAT agar at 30°C for 7 days. Bars, 10 μm. (**a**) Wild-type strain. (**b**) Δ*sipA* strain. (**c**) Δ*ssdA* strain. (**d**) Δ*sipA*Δ*ssdA* strain. (**e**) Δ*rsdK*Δ*rsdR* strain.

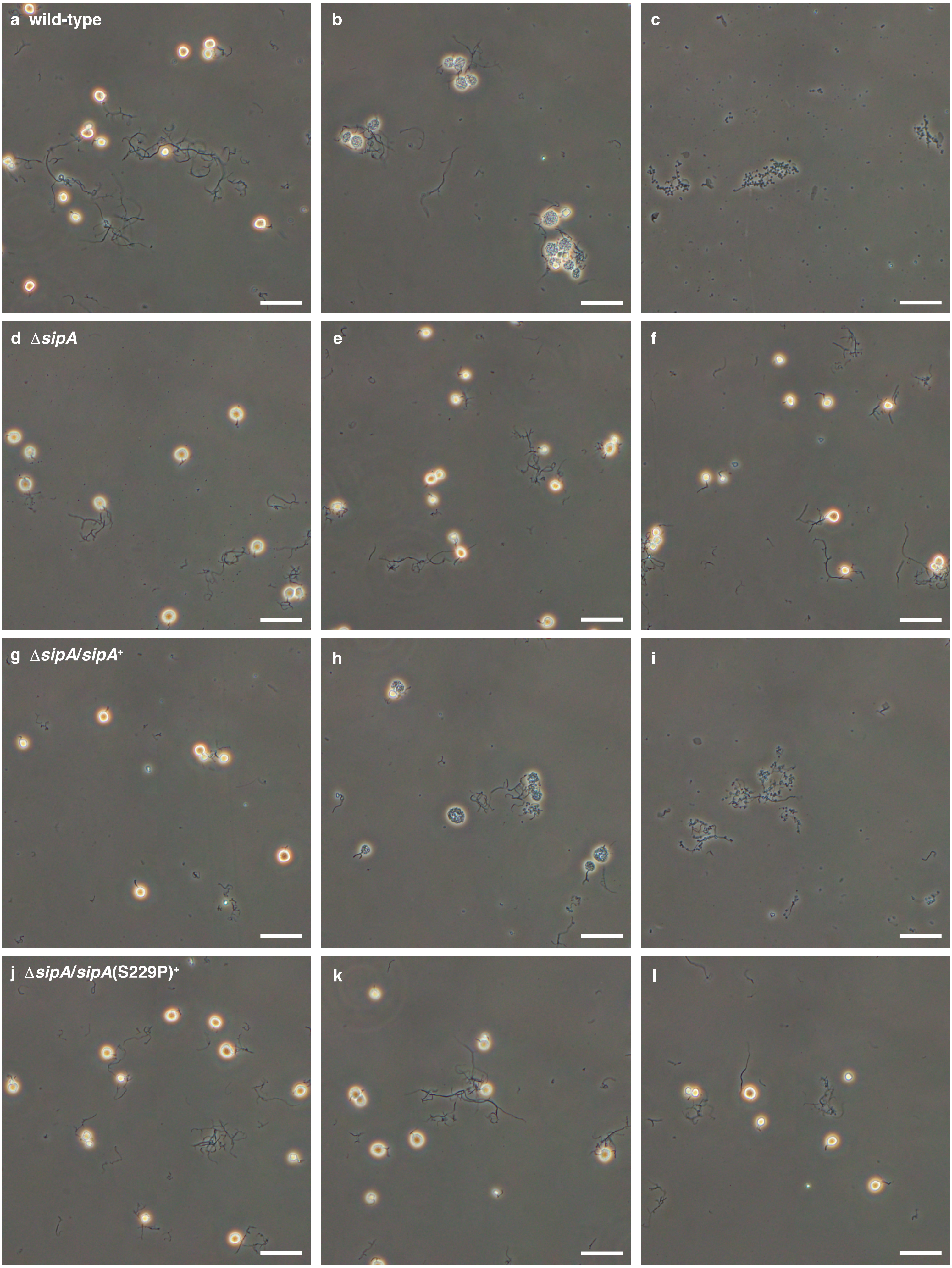

a wild-type
b
c
d ΔsipA
e
f
g ΔsipA/sipA+
h
i
j ΔsipA/sipA(S229P)+
k
l

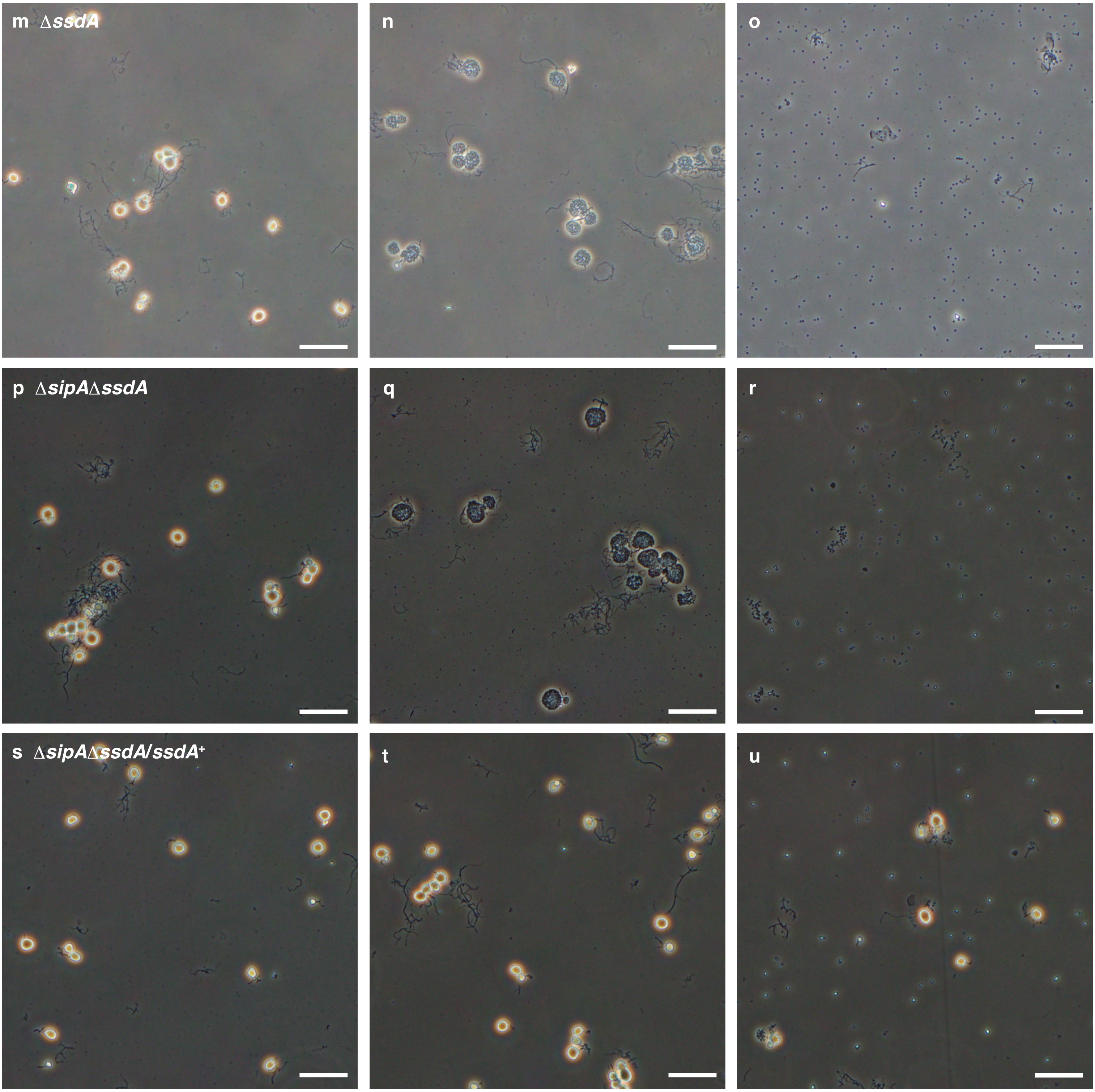

Fig. S4. Observation of sporangium dehiscence using phase-contrast microscopy. Sporangia produced on HAT agar were harvested and suspended in 25 mM histidine solution to induce sporangium dehiscence. Panels **a**–**u** are the entire images of the microscopic fields shown in Fig. 1a-u. Scale bars, 20 μm.

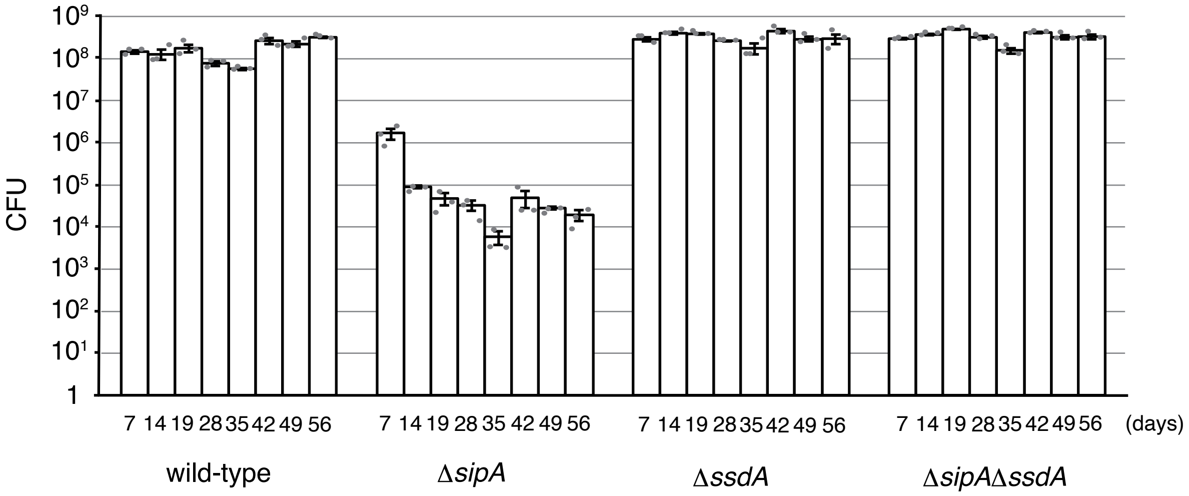

Fig. S5. Number of spores released from the sporangia after prolonged incubation. Wild-type, Δ*sipA*, Δ*ssdA*, and Δ*sipA*Δ*ssdA* strains were cultivated on HAT agar at 30°C for 7, 14, 19, 28, 35, 42, 49, and 56 days. Zoospores released from the sporangia formed on one HAT agar plate by pouring 25 mM NH_4HCO_3 solution were counted as CFU on YBNM agar. Data are means of three biological replicates ± standard error. Source data are provided as a Source Data file.

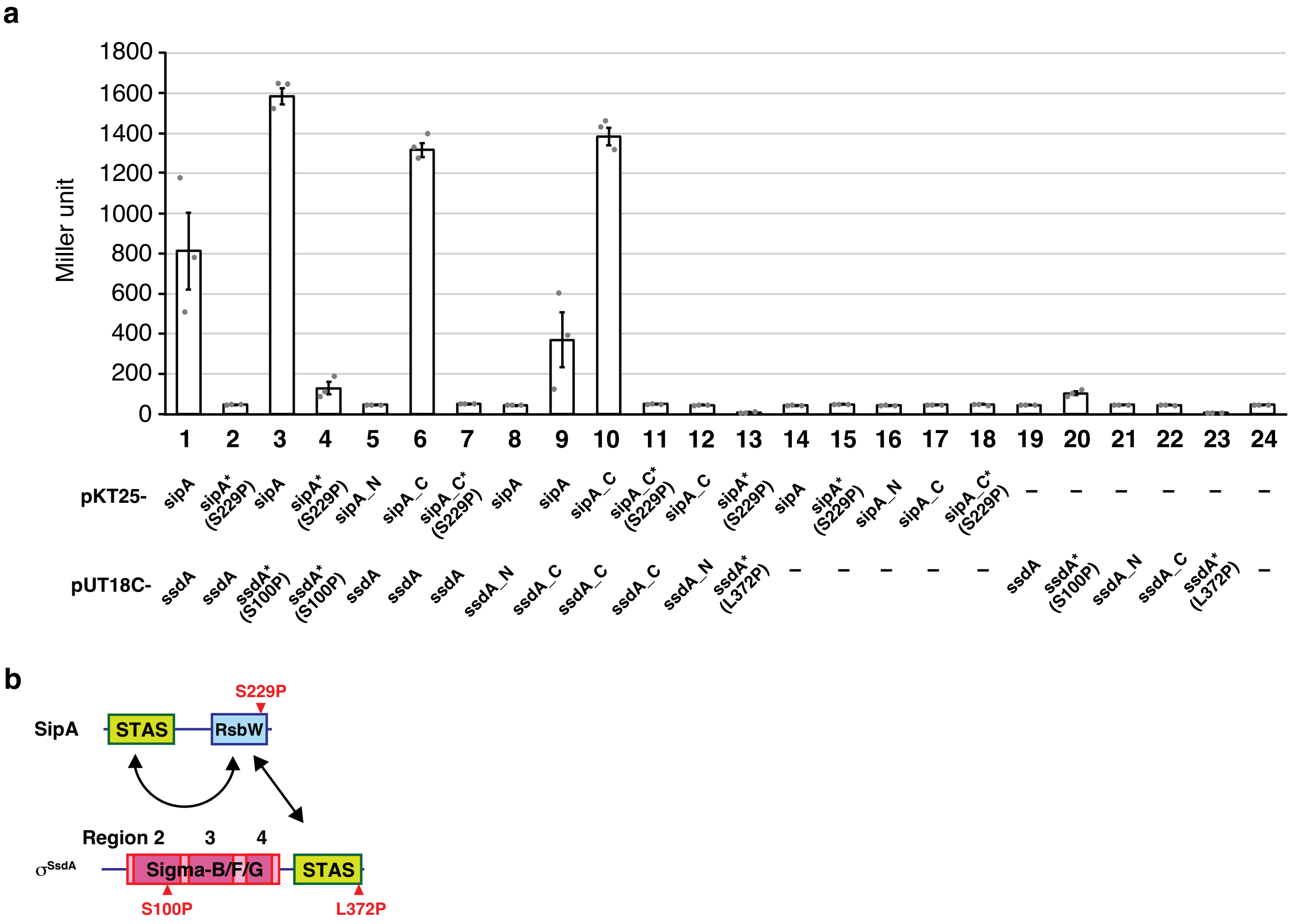

Fig. S6. BACTH assays for SipA and σ^{SsdA}. (**a**) β-Galactosidase activities (Miller units) of *E. coli* BTH101 co-transformed with two plasmids individually directing the production of full-length or truncated forms of SipA and σ^{SsdA}. The *sipA*_N and *sipA*_C genes encode truncated forms of the anti-sigma factor antagonist and anti-sigma factor domains of SipA, respectively. The *ssdA*_N and *ssdA*_C genes encode truncated forms of the sigma factor and anti-sigma factor antagonist domains of σ^{SsdA}, respectively. Plasmids for the production of the SipA (S229P), σ^{SsdA} (S100P), and σ^{SsdA} (L372P) variants were also used. The mutated genes are shown with asterisks. Empty vectors producing only the T18 and T25 domains of adenylate cyclase were used as vector controls. Values represent mean ± standard error of three biological replicates for each interaction. Source data are provided as a Source Data file. (**b**) Schematic of the domain structures in SipA and σ^{SsdA}. The domain combinations whose interactions were detected in the BACTH assays are indicated by double-headed arrows. The locations of S229P replacement in SipA and S100P and L372P replacement in σ^{SsdA} are indicated by red arrowheads.

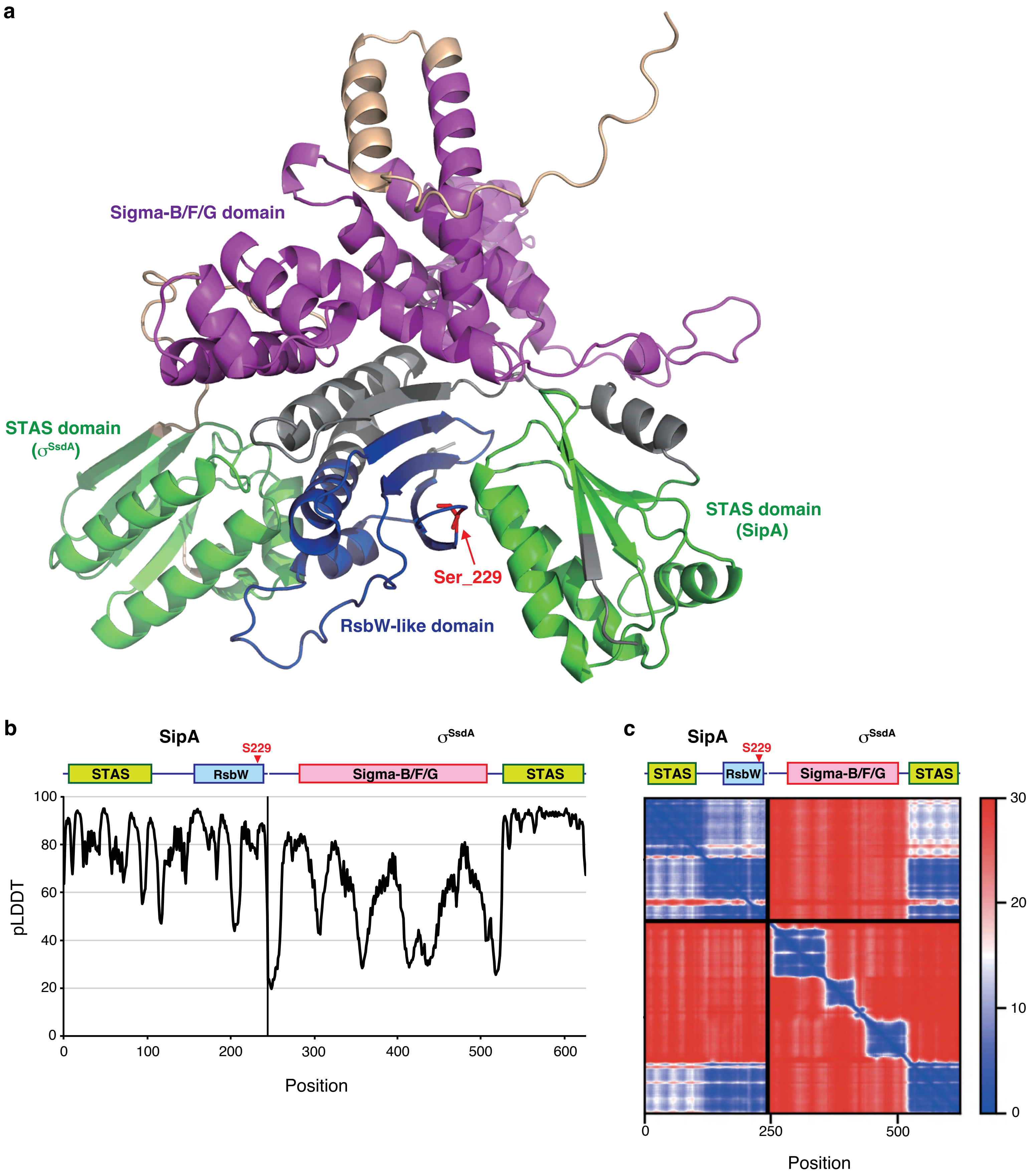
a
Sigma-B/F/G domain
STAS domain
(σ^{SsdA})
STAS domain
(SipA)
Ser_229
RsbW-like domain
b
SipA
σ^{SsdA}
S229
STAS
RsbW
Sigma-B/F/G
STAS
pLDDT
Position
c
SipA
σ^{SsdA}
S229
STAS
RsbW
Sigma-B/F/G
STAS
Position

d

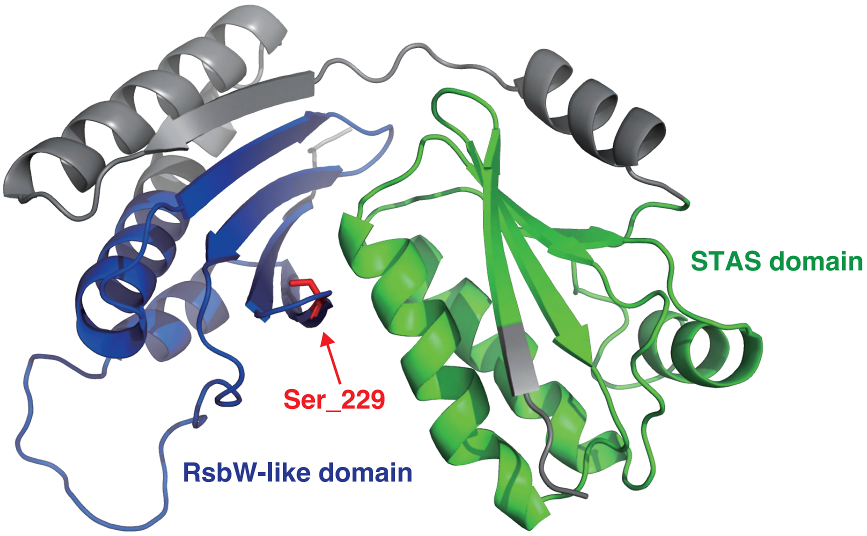
STAS domain
Ser_229
RsbW-like domain

e

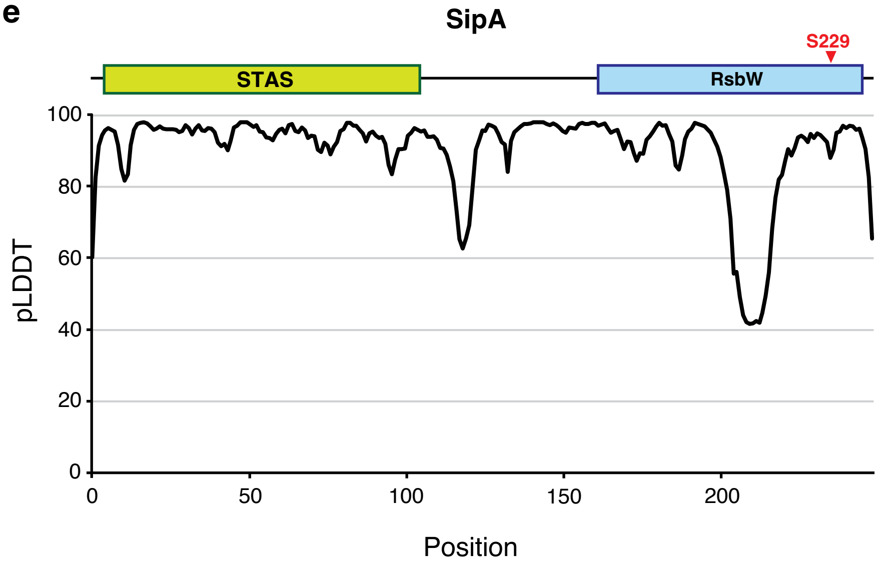
SipA
S229
STAS
RsbW
pLDDT
100
80
60
40
20
0
0
50
100
150
200
Position

f

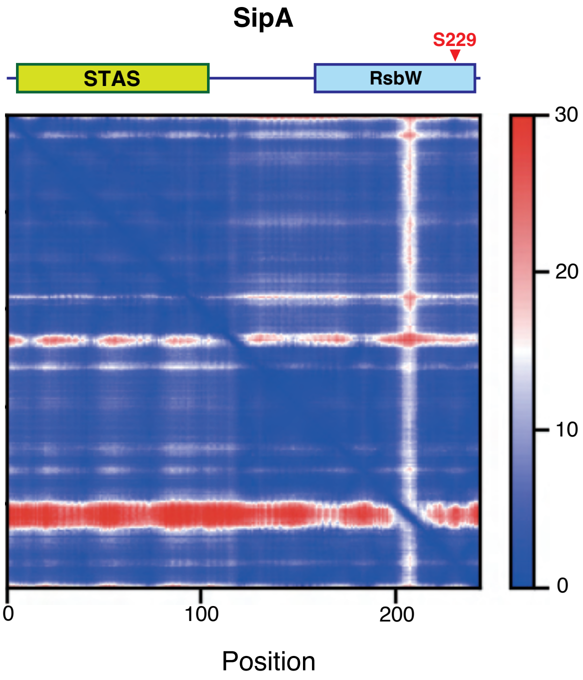
SipA
S229
STAS
RsbW
30
20
10
0
0
100
200
Position

g

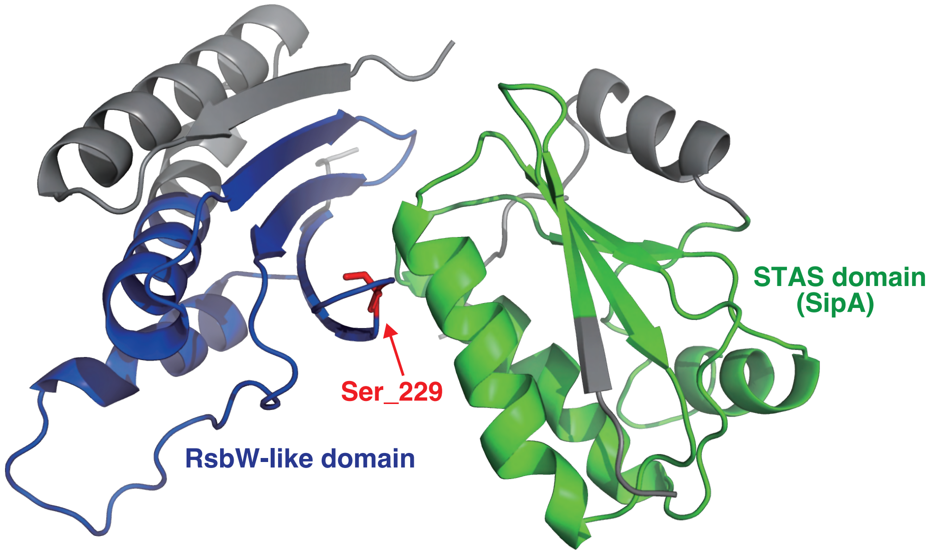

STAS domain
(SipA)
Ser_229
RsbW-like domain

h

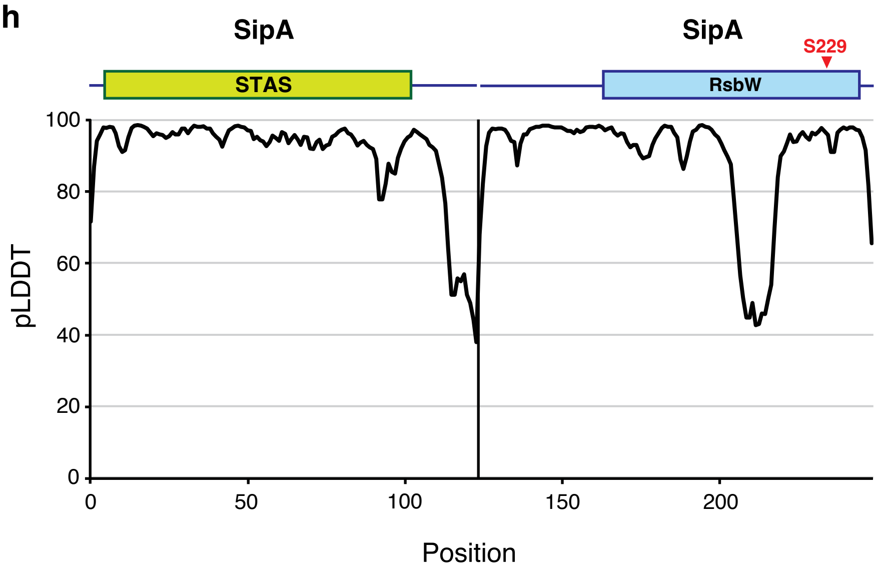

SipA
SipA
S229
STAS
RsbW
pLDDT
100
80
60
40
20
0
0
50
100
150
200
Position

i

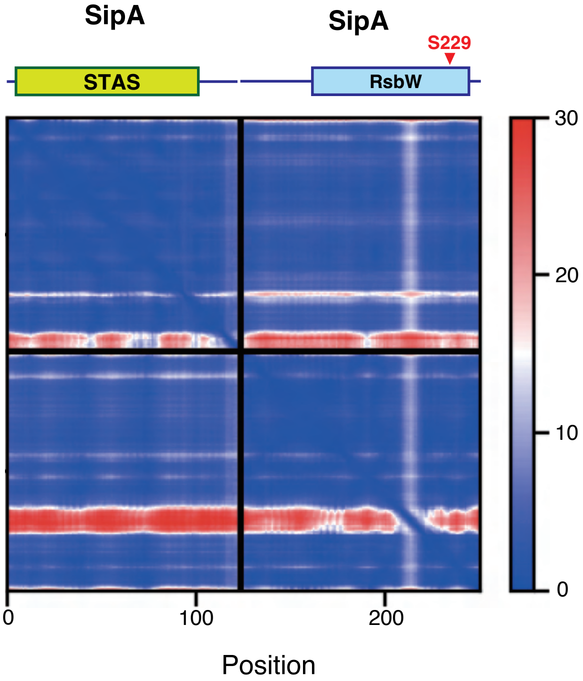

SipA
SipA
S229
STAS
RsbW
30
20
10
0
0
100
200
Position

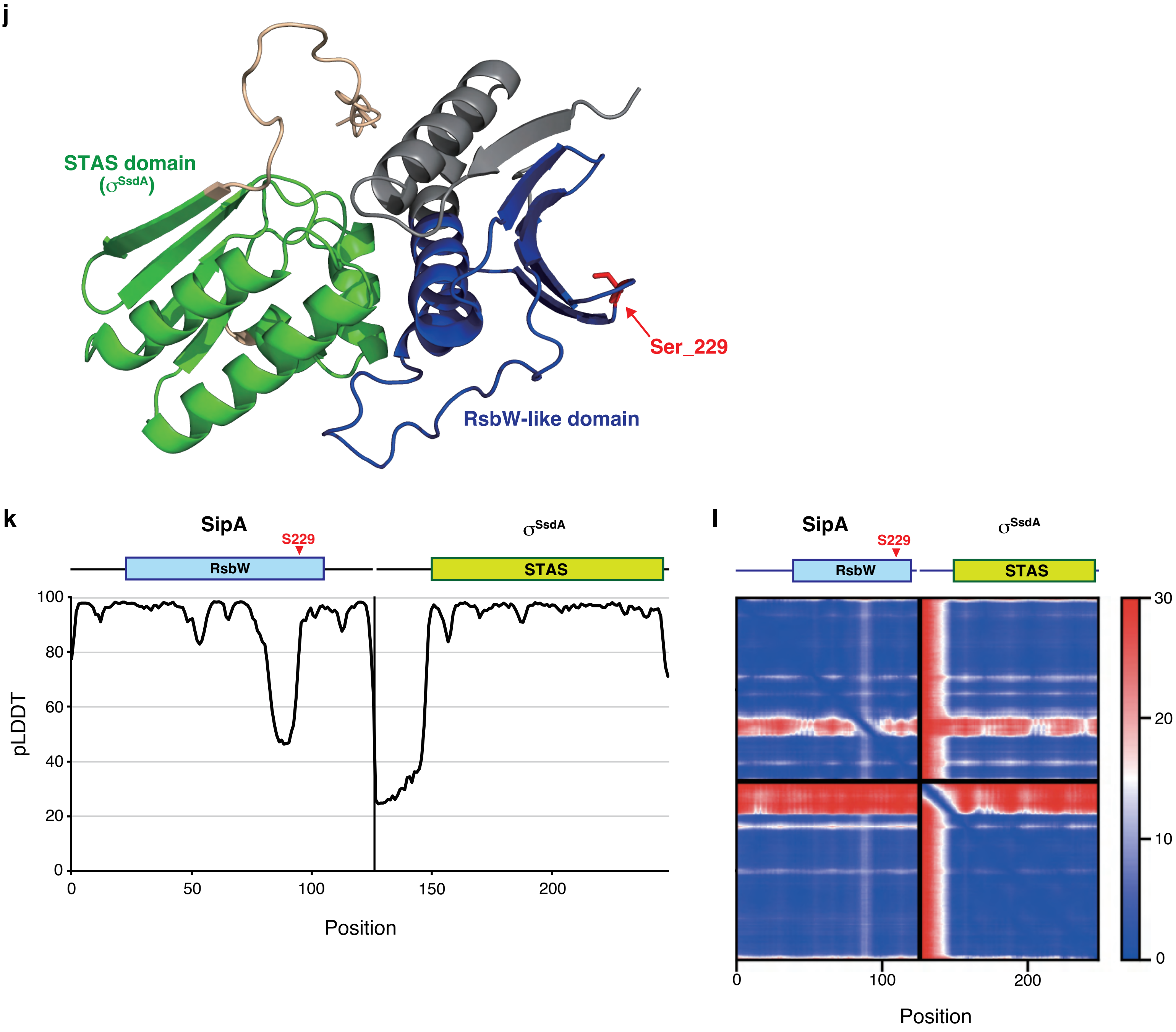
j
STAS domain
(σSsdA)
Ser_229
RsbW-like domain
k
SipA
σSsdA
S229
RsbW
STAS
pLDDT
100
80
60
40
20
0
0
50
100
150
200
Position
l
SipA
σSsdA
S229
RsbW
STAS
30
20
10
0
0
100
200
Position

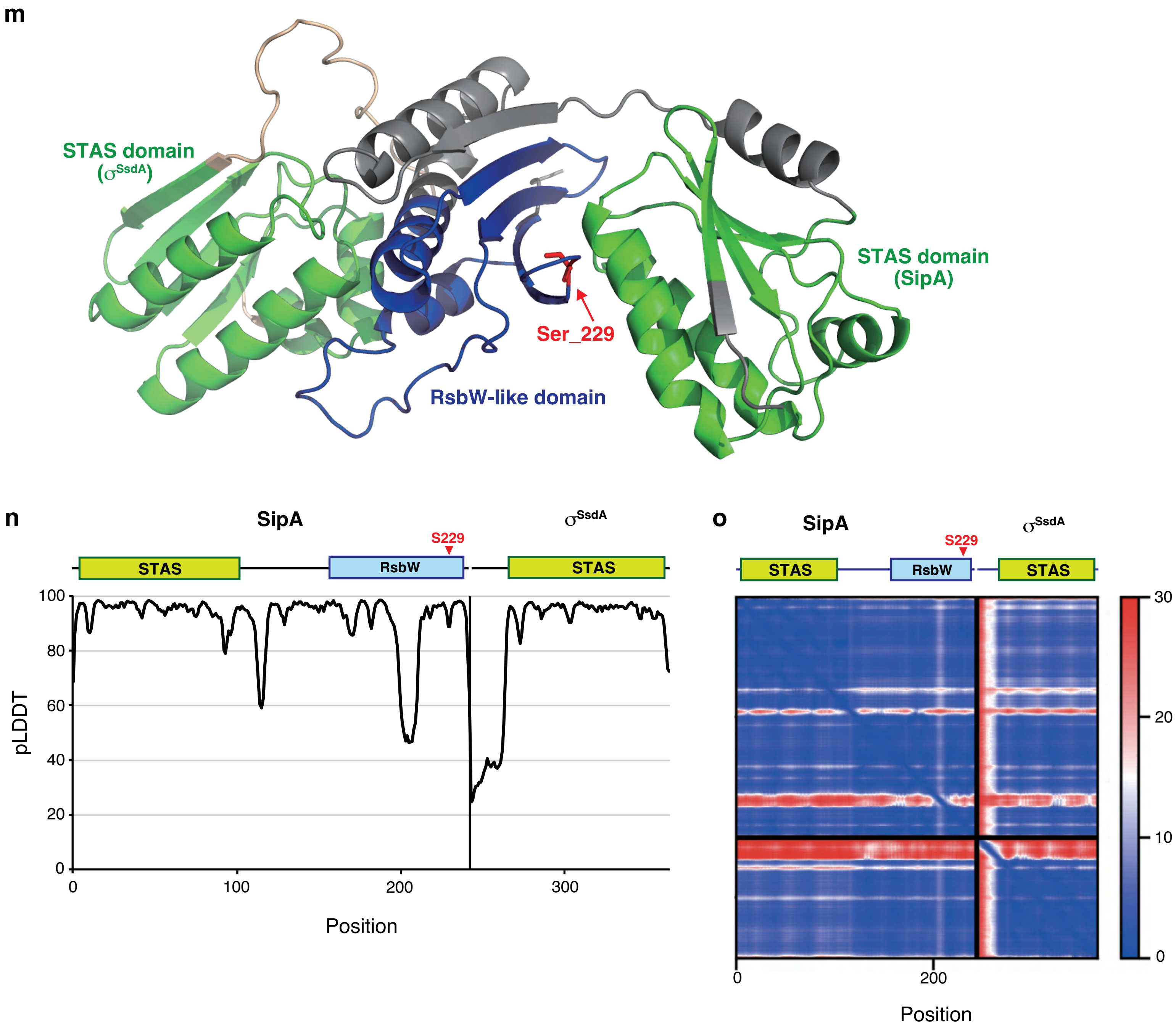

Fig. S7. AlphaFold- or AlphaFold-Multimer-based prediction of the SipA or SipA-σ^{SsdA} complex structures. Predicted structures of the heterodimer complex composed of full-length SipA and σ^{SsdA} (**a**), full-length SipA (**d**), a complex composed of the separate RsbW-like and STAS domains of SipA (**g**), a heterodimer complex composed of the RsbW-like domain of SipA and the STAS domain of σ^{SsdA} (**j**), and a heterodimer complex composed of full-length SipA and the STAS domain of σ^{SsdA} (**m**). Polypeptides are shown by ribbon representation and coloured green for the STAS domains (SipA and σ^{SsdA}), blue for the RsbW-like domain (SipA), and magenta for the sigma-B/F/G domain (σ^{SsdA}). The remaining residues of SipA and σ^{SsdA} are coloured grey and pale orange, respectively. Ser-229 in SipA is indicated by a red arrow. The predicted local distance difference test (pLDDT) (**b**, **e**, **h**, **k**, and **n**) and predicted aligned error (PAE) (**c**, **f**, **i**, **l**, and **o**) scores are shown below the structures. The pLDDT score, ranging between 0 and 100, characterizes local structural accuracy. The PAE score corresponds to topological accuracy between each residue of the complex. PDB files for predicted structures are available in Supplementary Data 1.

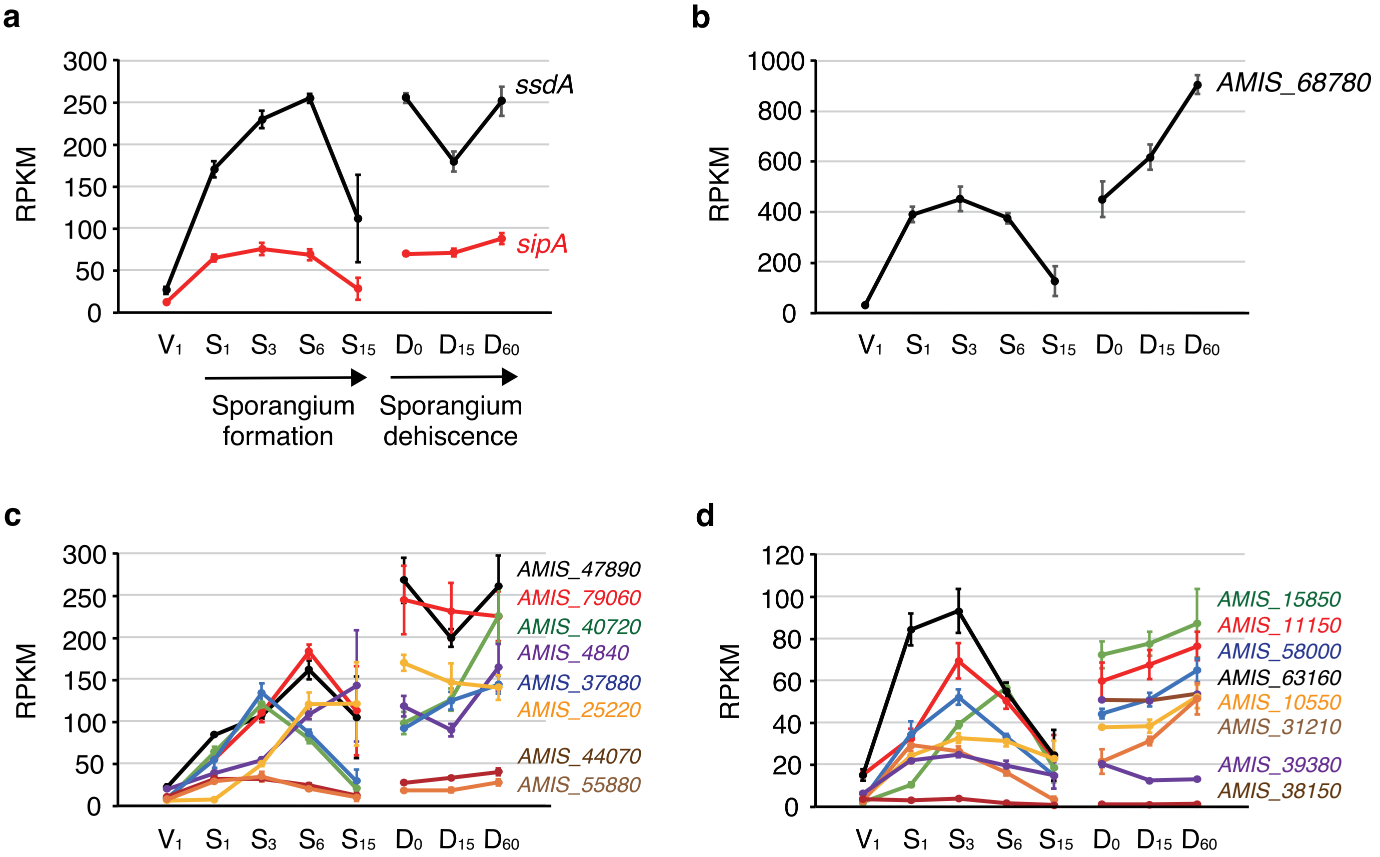

Fig. S8. Transcript levels of *sipA*, *ssdA* (a), and 17 genes under the direct control of σ^{SsdA}-recognizing promoters (b–d). Transcripts were examined using RNA-Seq analysis under various culture conditions. RNA samples were prepared from substrate hyphae grown on YBNM agar for 1 day (V_1), substrate hyphae or mixtures of substrate hyphae and sporangia grown on HAT agar for 1, 3, 6, and 15 days (S_1, S_3, S_6, and S_{15}, respectively), and sporangia (including some substrate hyphae) incubated in 25 mM histidine solution to induce sporangium dehiscence for 0, 15, and 60 min (D_0, D_{15}, and D_{60}, respectively). Average RPKM values ± standard errors for the three biological replicates are shown. Source data are provided as a Source Data file.

AMIS_4840	TTCGCAGGAATGGTTTCGGCCGATC-GGGGCATCCCGCCTGCATGC
AMIS_10550	GGCGCGGGTTTGGCAGCCGCAGGGT-CGGGCAACCGTGGTCTCGGA
AMIS_11150	CACATCCGGTTATCCGCACCTGATC-CGGGAAAGCGGATCGGGACC
AMIS_15850	GCATGGCGTTTCTTCGACCGGGACG-CGGGTACCCCCACACGCATG
AMIS_25220	TTGCGGGACATATCCGGCGTCGGGT-GGGGTAATCGCGCTGCTCCA
AMIS_31210	TGATCGCGGATAGCACCGAGGCTCG-CGGGTATCCCGGTGTCATGA
AMIS_37880	CGCAGACGTTCCGTCCGGTTTTTCG-TGGGTATGCAGGGCATCGCG
AMIS_38150	CCTCGACGGTAGAAACCGTCCGCCA-CGGGTAATCAGCTGATCATG
AMIS_39380	GCCCGGGGTTTGGTGGGGCACTCCGCCGGGTAGCCACGGCGCCGT
AMIS_40720	CCCGTGGGATTGATCGGTGGTTGAA-CGGGCAAGCAGACATCATGC
AMIS_44070	GGATCACGCTTCGAGCGGCACCGAT-CGGGTATGCACCGGACACCC
AMIS_47890	AGAAGAAGTGATTACCGGCTGCCGG-CGGGTACTCCTCAGGATGCC
AMIS_55880	CCGGAGTGTTTCCACGGCGCTGAACAGGGGCACCCTCCCGGCGTG
AMIS_58000	GATACGCGCATATTCGCCGCCCGGC-CGGGTAGGCGGCAGAGCGTC
AMIS_63160	ATCCGCCGCATGTTCGGCGGCAAATAGGGGTAACGGGCGGCCCAT
AMIS_68780	CACCCACGTTTACCGGCGGCCGGGC-GGGGCAATCGCGGCCCATGA
AMIS_79060	GTCATATGGTTTCCACGCAAGGATT-CGGGTACGCAGCGCCGCGCT
	GnTT -----n_{14-15}----- CGGGTA

Fig. S9. Nucleotide sequences of 17 σ^{SsdA}-recognizing promoter-containing regions. Bent arrows indicate the transcriptional start points predicted by our unpublished dRNA-Seq analysis. Putative σ^{SsdA}-recognizing promoters are shown in red. The consensus σ^{SsdA}-recognizing promoter sequence is shown at the bottom of the figure.

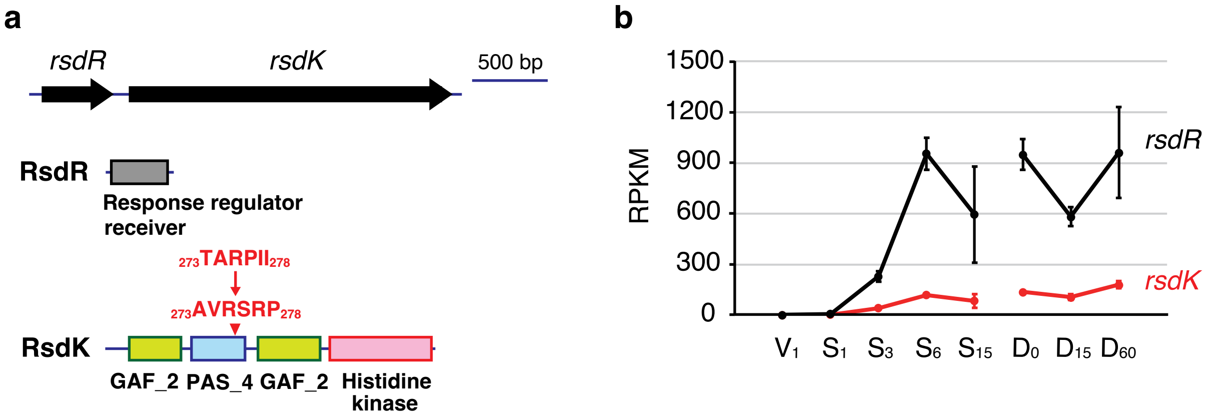

Fig. S10. Schematic representation of the *rsdR*-*rsdK* operon and domains of RsdK and RsdR (a) and transcript levels of *rsdK* and *rsdR* (b). (**a**) The location of the amino acid replacement in RsdK generated in strain S-7 is indicated by a red arrowhead. (**b**) Transcripts were examined using RNA-Seq, as described in the legend of Fig. S8. Average RPKM values ± standard errors for the three biological replicates are shown. Source data are provided as a Source Data file.

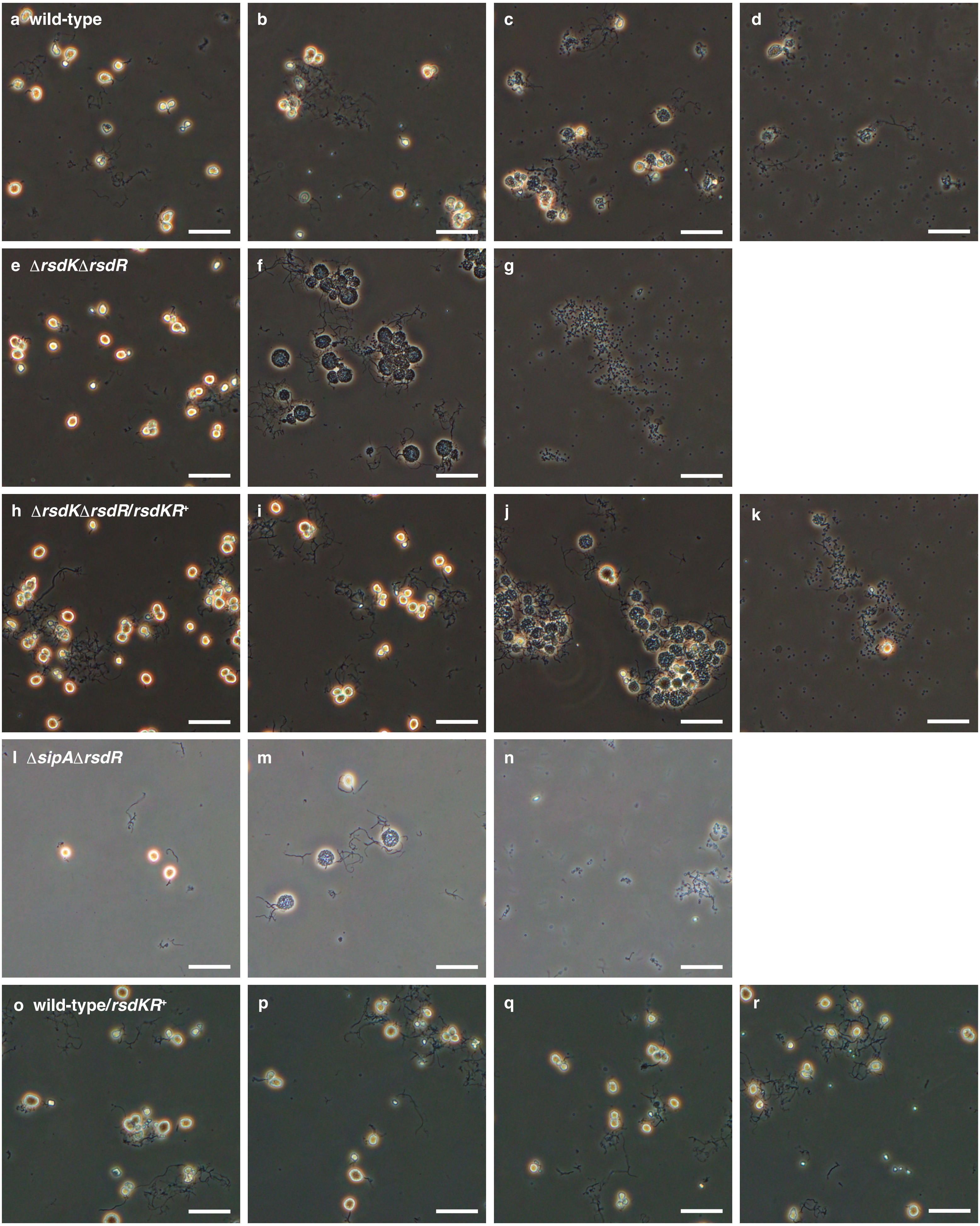

Fig. S11. Observation of sporangia and zoospores using phase-contrast microscopy. Sporangia produced on HAT agar were harvested and suspended in 25 mM histidine solution to induce sporangium dehiscence. Panels **a**–**r** are the entire images of the microscopic fields shown in Fig. 4a–r. Scale bars, 20 μm.

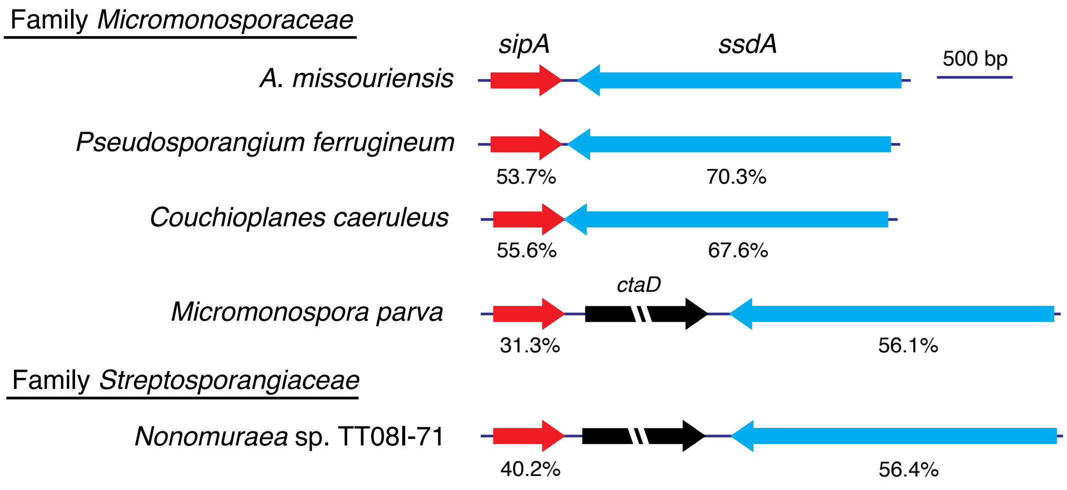

Fig. S12. Distribution of *sipA* and *ssdA* homologues in actinomycetes. In members of the genera *Actinoplanes*, *Pseudosporangium*, and *Couchioplanes*, *sipA* homologues are located on the gene locus adjacent to *ssdA* homologues. In species of the genus *Micromonospora*, a gene encoding cytochrome c oxidase subunit I (*ctaD*) is located between the *sipA* and *ssdA* homologues. In species other than members of the family *Micromonosporaceae* (to which all the genera described above belong), *Nonomuraea* sp. TT08I-71, which belongs to the family *Streptosporangiaceae*, has *sipA* and *ssdA* homologues, between which a *ctaD* homologue is also located.

Table S1. Mutations identified in the sporangium dehiscence-deficient mutant strains[a]

Strain ID	Genomic position	Gene ID[c]	Gene product	Reference[d]	Allele[d]	Mutation type[e]	Depth[f]	Frequency (%)[g]
M-1[b]	232,976	AMIS_2080	putative iron ABC transporter substrate-binding protein	A	G	SNV	45	100
	854,063	AMIS_8000	putative amidase	A	G	SNV	62	100
	2,215,545	AMIS_21050	hypothetical protein	T	C	SNV	60	98
	7,595,196	AMIS_70090	hypothetical protein	C	T	SNV	58	100
	8,273,443	AMIS_76580	Response regulator (TcrA)	A	C	SNV	52	100
M-2[b]	518,019	AMIS_4800	putative LacI-family transcriptional regulator	G	C	SNV	25	100
	836,785	Intergenic	-	T	A	SNV	79	100
	6,984,866-6,984,867	AMIS_64930	Hybrid sensor histidine kinase (HhkA)	GT	AC	MNV	46	100
	6,984,872			G	A	SNV	49	100
	7,858,397	AMIS_72460	hypothetical protein	A	T	SNV	52	100
M-3[b]	2,838,060	AMIS_26970	hypothetical protein	A	C	SNV	47	100
	3,820,466	AMIS_36530	putative diguanylate cyclase/phosphodiesterase	C	T	SNV	70	100
	4,771,834	AMIS_45590	putative ABC transporter ATP-binding protein	C	T	SNV	42	100
	6,984,245-6,984,246	AMIS_64930	Hybrid sensor histidine kinase (HhkA)	GA	CT	MNV	106	100
M-4[b]	2,838,060	AMIS_26970	hypothetical protein	A	C	SNV	32	100
	3,820,466	AMIS_36530	putative diguanylate cyclase/phosphodiesterase	C	T	SNV	53	100
	4,771,834	AMIS_45590	putative ABC transporter ATP-binding protein	C	T	SNV	22	100
	4,994,445	AMIS_47620	hypothetical protein	C	G	SNV	46	100
	6,984,245-6,984,246	AMIS_64930	Hybrid sensor histidine kinase (HhkA)	GA	CT	MNV	59	100
M-5[b]	1,464,196	AMIS_13680	hypothetical protein	T	A	SNV	57	100
	6,432,207	AMIS_60090	putative glycosyltransferase	T	A	SNV	46	100
	6,983,857-6,983,859	AMIS_64930	Hybrid sensor histidine kinase (HhkA)	CCT	-	Deletion	50	68
	8,763,182	Intergenic	-	T	C	SNV	29	100
M-6[b]	1,464,196	AMIS_13680	hypothetical protein	T	A	SNV	51	100
	6,432,207	AMIS_60090	putative glycosyltransferase	T	A	SNV	40	98
	6,983,857-6,983,859	AMIS_64930	Hybrid sensor histidine kinase (HhkA)	CCT	-	Deletion	37	65
	8,763,182	Intergenic	-	T	C	SNV	25	96
M-7[b]	1,169,757	AMIS_10960	putative globin-like protein	A	G	SNV	91	100
	1,464,196	AMIS_13680	hypothetical protein	T	A	SNV	94	100
	6,432,207	AMIS_60090	putative glycosyltransferase	T	A	SNV	89	100
	6,983,857-6,983,859	AMIS_64930	Hybrid sensor histidine kinase (HhkA)	CCT	-	Deletion	87	72
	7,176,832	AMIS_66410	putative glycosyltransferase	T	C	SNV	44	100
	8,763,182	Intergenic	-	T	C	SNV	40	100
M-8[b]	1,464,196	AMIS_13680	hypothetical protein	T	A	SNV	56	100
	6,432,207	AMIS_60090	putative glycosyltransferase	T	A	SNV	50	100
	6,708,691	Intergenic	-	G	C	SNV	24	100
	6,983,857-6,983,859	AMIS_64930	Hybrid sensor histidine kinase (HhkA)	CCT	-	Deletion	59	66
	8,763,182	Intergenic	-	T	C	SNV	21	100
M-9[b]	1,464,196	AMIS_13680	hypothetical protein	T	A	SNV	56	100
	4,044,901	AMIS_38570	putative MFS transporter	G	G	SNV	10	50
	6,432,207	AMIS_60090	putative glycosyltransferase	T	A	SNV	39	100
	6,983,857-6,983,859	AMIS_64930	Hybrid sensor histidine kinase (HhkA)	CCT	-	Deletion	45	76
	8,763,182	Intergenic	-	T	C	SNV	17	100
M-10[b]	289,423	Intergenic	-	C	A	SNV	203	51
	289,425	Intergenic	-	C	G	SNV	184	50
	1,319,281	Intergenic	-	G	A	SNV	119	100
	2,864,879	AMIS_27210	putative FMN-dependent S-2-hydroxy-acid dehydrogenase	T	C	SNV	78	99
	6,232,485	AMIS_58230	hypothetical protein	G	A	SNV	14	100
	6,983,868	AMIS_64930	Hybrid sensor histidine kinase (HhkA)	A	G	SNV	75	100
	7,491,944-7,491,945	AMIS_69160	putative serine/threonine protein kinase	TT	TC	MNV	53	100
M-11	6,456,889	AMIS_60200	putative DNA helicase	C	G	SNV	29	100
	6,488,854	AMIS_60400	putative MFS transporter	A	G	SNV	30	100
M-12	5,765,045	AMIS_54230	hypothetical protein (SipA)	T	C	SNV	56	100
M-13	884,335	AMIS_8240	putative ABC transporter substrate-binding protein	A	C	SNV	25	100
	1,256,323	AMIS_11710	putative enoyl-CoA hydratase/isomerase	T	G	SNV	29	100
	1,507,334	AMIS_14020	putative ABC transporter ATP-binding protein	C	T	SNV	31	100
	1,509,427-1,509,428	AMIS_14060	hypothetical protein	AT	TC	MNV	34	97
	2,655,749	Intergenic	-	A	T	SNV	25	100
	2,655,753	Intergenic	-	T	TG	Insertion	24	88
	2,739,612	AMIS_26140	hypothetical protein	A	C	SNV	36	100
	2,950,106-2,950,107	AMIS_28050	putative ABC transporter permease protein	AA	CT	MNV	30	100
	7,115,313	AMIS_65960	putative glutamate-cysteine ligase	T	C	SNV	23	100
M-14	52,158	AMIS_470	putative serine/threonine protein kinase	C	T	SNV	43	100
	2,116,943	AMIS_19360	putative SARP-family transcriptional activator	G	A	SNV	43	100
	2,556,626	AMIS_24430	hypothetical protein	G	A	SNV	86	100
	3,168,736	AMIS_30150	hypothetical protein	C	T	SNV	81	99
	4,064,583	AMIS_38780	putative secreted protein containing F5/8 type C domain	T	G	SNV	66	100

M-15	5,888,563	AMIS_55270	putative two-component system sensor kinase	T	C	SNV	78	97
	6,151,110	AMIS_57480	putative acyl-CoA synthetase	A	G	SNV	105	100
	7,770,469	AMIS_71640	putative isobutyryl-CoA mutase chain A	C	T	SNV	75	99
	7,808,421	AMIS_72010	putative serine hydroxymethyltransferase	G	C	SNV	43	100
	7,831,717-7,831,718	AMIS_91460	tRNA-Arg	GC	G	Deletion	46	83
	8,508,671	AMIS_78700	putative sensor-like histidine kinase	C	T	SNV	33	100
M-16[b]	494,983	Intergenic	-	G	C	SNV	43	100
	1,657,953	AMIS_15380	putative DNA polymerase III α subunit	C	T	SNV	54	100
	1,864,317	AMIS_17220	putative indolepyruvate decarboxylase	G	A	SNV	18	100
	3,145,104	AMIS_29880	hypothetical protein	T	C	SNV	14	100
	6,983,857-6,983,859	AMIS_64930	Hybrid sensor histidine kinase (HhkA)	CCT	-	Deletion	32	59
	8,565,276	AMIS_79250	putative iron ABC transporter ATP-binding protein	G	A	SNV	29	100
M-17	392,849	AMIS_3730	hypothetical protein	G	A	SNV	60	100
	568,933	AMIS_5210	putative inositol monophosphatase	C	A	SNV	60	100
	2,354,811	AMIS_22690	putative MFS transporter	C	T	SNV	45	100
	2,877,938	AMIS_27350	putative TetR-family transcriptional regulator	T	C	SNV	66	100
	5,154,303	AMIS_49020	putative hydrolase	A	G	SNV	53	94
	7,200,073	AMIS_66600	hypothetical protein	C	T	SNV	48	100
	8,228,193	AMIS_76070	putative response regulator receiver domain protein (AsfR)	C	G	SNV	53	100
	8,538,514	Intergenic	-	A	G	SNV	85	99
M-18[b]	1,130,270	AMIS_10600	putative MFS transporter	C	A	SNV	49	100
	6,575,146-6,575,147	AMIS_61150	putative phosphoenolpyruvate-dependent sugar phosphotransferase	GG	TA	MNV	47	100
	6,979,706-6,992,297	-	-	-	-	Deletion	-	-
	7,344,392	AMIS_67730	putative cholesterol oxidase	G	A	SNV	15	100
	8,456,699	AMIS_78220	putative adenylate/guanylate cyclase	C	T	SNV	33	100
M-19	2,029,505	AMIS_18610	putative multi-sensor signal transduction histidine kinse	T	G	SNV	49	100
M-20	614,394-614,395	AMIS_5640	putative NADH dehydrogenase chain I	CC	TT	MNV	50	100
	2,032,592	AMIS_18620	putative multi-sensor signal transduction histidine kinse	G	A	SNV	36	100
	4,120,369-4,120,370	AMIS_39280	putative LuxR-family transcriptional regulator	GT	AA	MNV	17	100
	4,312,761	AMIS_40810	putative GCN5-related N-acetyltransferase	T	C	SNV	11	100
	6,267,788	AMIS_58560	putative AfsR-family transcriptional regulator	G	A	SNV	21	100
	7,118,461	AMIS_65980	putative peptidase	C	G	SNV	33	100
M-21	1,734,290	AMIS_16060	putative excinuclease ABC subunit C	C	T	SNV	38	100
	5,372,885	AMIS_50690	putative ABC transporter ATPase and permease protein	G	C	SNV	30	100
M-22	805,058	AMIS_7490	putative glycosyltransferase	A	G	SNV	40	100
	6,714,616	AMIS_62440	hypothetical protein	G	A	SNV	11	55
	6,810,519	AMIS_63330	putative LuxR-family transcriptional regulator	G	A	SNV	31	100
	7,362,061	AMIS_67920	putative ATP-dependent dsDNA exonuclease C subunit	C	T	SNV	44	100
	8,447,148-8,447,149	AMIS_78170	putative capsule synthesis protein	CC	TCT	Insertion, SNV	29	69
M-23	5,470,230	AMIS_51490	putative ABC transporter permease protein	G	A	SNV	78	100
M-24	5,095,834	AMIS_48500	putative ABC transporter ATPase and permease protein	T	C	SNV	35	100
	6,603,482	AMIS_61430	putative glycoside hydrolase	C	T	SNV	29	100
M-25[b]	1,427,164	AMIS_13370	hypothetical protein	A	T	SNV	48	100
	1,494,443	AMIS_13920	putative glycine cleavage system T protein	C	T	SNV	74	100
	4,408,436	AMIS_41960	putative polypeptide deformylase	G	C	SNV	43	100
	5,247,370	AMIS_49640	putative NRPS	C	T	SNV	51	100
	5,247,397			C	A	SNV	41	98
	5,247,402			C	T	SNV	35	97
	5,247,537			T	A	SNV	44	59
	5,732,437	AMIS_53920	hypothetical protein	G	A	SNV	49	98
	6,984,828	AMIS_64930	Hybrid sensor histidine kinase (HhkA)	C	T	SNV	90	100
	6,985,785	AMIS_64940	putative ribose-phosphate pyrophosphokinase	C	T	SNV	62	100
M-26[b]	384,225	AMIS_3650	putative tryptophan synthase α chain	T	C	SNV	40	100
	2,557,522	AMIS_24440	putative FAD-dependent oxidoreductase	A	G	SNV	29	100
	5,697,992	Intergenic	-	T	C	SNV	58	100
	7,974,231	Intergenic	-	A	G	SNV	32	100
	8,273,234	AMIS_76580	Response regulator (TcrA)	G	A	SNV	37	100
M-27	662,218	AMIS_6180	putative ribosomal protein L6	C	G	SNV	90	99
	1,476,890	AMIS_13770	putative AAA ATPase	A	G	SNV	89	100

[a] Mutations identified in the wild-type strain, whose genome sequence was analyzed in parallel, were eliminated.

[b] Mutations within *hhkA* and *tcrA* are shown in orange and yellow, respectively.

[c] Gene identifiers are shown in case mutations were identified in coding sequences. Mutations identified in intergenic regions are shown as "Intergenic".

[d] Nucleotides on the wild-type and mutant genomes are shown in the "Reference" and "Allele" columns, respectively.

[e] Mutations of single and multiple nucleotide variants are shown as "SNV" and "MNV", respectively.

[f] Counts of the sequence reads on the mutation points are shown.

[g] Proportion of the mutated read counts to total read counts on the mutation points are shown.

Table S2. Mutations identified in the sporangium dehiscence-competent suppressor strains[a]

Strain ID	Genomic position	Gene ID[c]	Gene product	Reference[d]	Allele[d]	Mutation type[e]	Depth[f]	Frequency (%)[g]
S-7[b]	1409320	Intergenic	-	GT	AC	MNV	104	98
S-7[b]	1409664	Intergenic	-	G	A	SNV	148	99
S-7[b]	2901410	AMIS_27580	putative secretory lipase	G	A	SNV	161	99
S-7[b]	3950278	AMIS_37680	putative multi-sensor signal transduction histidine kinase (RsdK)	A	G	SNV	19	100
S-7[b]	3950282	AMIS_37680	putative multi-sensor signal transduction histidine kinase (RsdK)	-	T	Insertion	22	100
S-7[b]	3950284	AMIS_37680	putative multi-sensor signal transduction histidine kinase (RsdK)	-	G	Insertion	22	100
S-7[b]	3950285	AMIS_37680	putative multi-sensor signal transduction histidine kinase (RsdK)	G	T	SNV	22	100
S-7[b]	3950290	AMIS_37680	putative multi-sensor signal transduction histidine kinase (RsdK)	A	-	Deletion	22	100
S-7[b]	3950293	AMIS_37680	putative multi-sensor signal transduction histidine kinase (RsdK)	AT	C	Replacement	25	100
S-7[b]	4047857	AMIS_38600	hypothetical protein	G	A	SNV	155	99
S-7[b]	4529269	AMIS_43200	putative tetracycline resistance protein	C	T	SNV	158	100
S-7[b]	5610925	AMIS_52810	putative glycoside hydrolase	-	TTGGTGCTGGCC	Insertion	93	98
S-7[b]	5610926	AMIS_52810	putative glycoside hydrolase	A	CGT	Replacement	92	98
S-7[b]	6155558	AMIS_57520	hypothetical protein	T	C	SNV	152	100
S-7[b]	8408431	AMIS_77840	hypothetical protein	C	T	SNV	141	99
S-9[b]	4431145	AMIS_42150	putative NAD synthetase	AC	TT	MNV	171	100
S-9[b]	5766033	AMIS_54240	putative RNA polymerase sigma factor with STAS domain (σ^{SsdA})	A	G	SNV	176	100
S-9[b]	5934550	AMIS_55660	putative ABC transporter permease protein	C	T	SNV	152	98
S-9[b]	7151360	AMIS_66220	putative ABC transporter ATP-binding protein	G	T	SNV	178	99
S-9[b]	7923832	AMIS_73080	putative inositol monophosphatase	C	T	SNV	195	99
S-14[b]	5766007	AMIS_54240	putative RNA polymerase sigma factor with STAS domain (σ^{SsdA})	A	G	SNV	158	99
S-20[b]	958330	AMIS_8950	hypothetical protein	G	A	SNV	148	99
S-20[b]	1298995	Intergenic	-	T	G	SNV	116	50
S-20[b]	2090035	AMIS_19070	hypothetical protein	A	G	SNV	175	99
S-20[b]	2558467	AMIS_24450	putative TetR-family transcriptional regulator	G	A	SNV	119	99
S-20[b]	3609491	Intergenic	-	-	A	Insertion	116	99
S-20[b]	3792842	Intergenic	-	A	G	SNV	173	100
S-20[b]	5206314	AMIS_49360	hypothetical protein	C	T	SNV	167	100
S-20[b]	5674071	AMIS_53330	putative AfsR-family transcriptional regulator	A	G	SNV	170	100
S-20[b]	5765190	AMIS_54240	putative RNA polymerase sigma factor with STAS domain (σ^{SsdA})	A	G	SNV	127	100
S-21[b]	276800	AMIS_2570	hypothetical protein	T	C	SNV	364	100
S-21[b]	1284347	AMIS_11990	hypothetical protein	G	A	SNV	178	99
S-21[b]	2786760	AMIS_26470	hypothetical protein	G	A	SNV	173	100
S-21[b]	3828695	AMIS_36590	hypothetical protein	A	G	SNV	201	100
S-21[b]	5506461	AMIS_51860	hypothetical protein	C	T	SNV	192	100
S-21[b]	5506465	AMIS_51860	hypothetical protein	T	C	SNV	189	100
S-21[b]	5766103	AMIS_54240	putative RNA polymerase sigma factor with STAS domain (σ^{SsdA})	C	T	SNV	157	99
S-21[b]	6841986	AMIS_63700	putative *O* -methyltransferase	C	T	SNV	153	99
S-21[b]	7292072	AMIS_67300	hypothetical protein	T	C	SNV	169	99
S-21[b]	7495125	AMIS_69170	putative ATP-dependent DNA helicase	C	T	SNV	154	99

[a] Mutations identified in the parental strain M-12, whose genome sequence was determined in parallel, were eliminated.

[b] Mutations within *ssdA* and *rsdK* are shown in orange and yellow, respectively.

[c] Gene identifiers are shown in case mutations were identified in coding sequences. Mutations identified in intergenic regions are shown as "Intergenic".

[d] Nucleotides on the parental strain M-12 and obtained suppressor genomes are shown in the "Reference" and "Allele" columns, respectively.

[e] Mutations of single and multiple nucleotide variants are shown as "SNV" and "MNV", respectively.

[f] Counts of the sequence reads on the mutation points are shown.

[g] Proportion of the mutated read counts to total read counts on the mutation points are shown.

Table S3. Primers used in this study

Primer name	Sequence (5' to 3')[a]	Used for
AMIS54230-UF1	GCCAAGCTTTGACGTACGGTAGCGTTGCCA	Gene disruption
AMIS54230-UR1	GCTCTAGAGAGACACTTGAGCAGGCTGT	Gene disruption
AMIS54230-DF1	GCTCTAGACTGTTGCTGGTCAGCTCGGT	Gene disruption
AMIS54230-UR1	GGAATTCGACCCACAGATCCCTGCAAC	Gene disruption
AMIS54240-UF2	GGGGTACCGTCGTCGTCCGGCATCATTG	Gene disruption
AMIS54240-UR2	TTTCCTGCAGGGCTCCAGGTCCTCCAGGTACT	Gene disruption
AMIS54240-DF2	TTTCCTGCAGGGTCGTGGTGGACTTCTCCGGT	Gene disruption
AMIS54240-DR2	CCCAAGCTTTATCTGCGATCCGACGTGCT	Gene disruption
AMIS37670-UF1	GGAATTCACCTGTGGATGCACTTCAC	Gene disruption
AMIS37670-UR2	CCATCGATGAACACCCTGGTCAGAACCAG	Gene disruption
AMIS37680-DF2	CCATCGATTTCTACCGGGGGAGCAATGTG	Gene disruption
AMIS37680-DR2	CGTCTAGAGCTCGACAACGACCTCATG	Gene disruption
AMIS54230-prm1-F1	GGAATTCAGGAGGTCACGGTCAGAGAT	Gene complementation
AMIS54230-prm1-R1	TTCTCTCCGACGGGGCCAGCCTTTCCCAGTT	Gene complementation
AMIS54230-prm1-F2	GCTGGCCCCGTCGGAGAGAAGGCGGAAATC	Gene complementation
AMIS54230-R1	GGCAAGCTTGGTGGATCTCTGCGTGAGCG	Gene complementation
AMIS54240-F1	GGAATTCCCTGACATGGGCCAGCACCA	Gene complementation
AMIS54240-R1	CCCAAGCTTCTGATCGTCACCGAGCTCGT	Gene complementation
AMIS_37670-37680-F1	GGAATTCTGGCATAGACCAGCTCACACT	Gene complementation
AMIS_37670-37680-R1	GCCAAGCTTGGCTTGCGGCACTGATCAA	Gene complementation
AMIS54230-ORF-F3	AACTGCAGGGATGGCGGTCCACTGCGACGT	BACTH assay
AMIS54230-ORF-R2	CGGGATCCTCCGGCTCGCGCCGGAGGGCGG	BACTH assay
sipA-TR1	CGGGATCCTCCTCCCGGATCACGGGATA	BACTH assay
sipA-TF1	AACTGCAGGGGTCATACCCGTGATCCGGGA	BACTH assay
sipA-TF3	AACTGCAGGGTCATACCCGTGATCCGGGA	BACTH assay
sipA-TF2	AACTGCAGGATGGCGGTCCACTGCGACGT	BACTH assay
AMIS54240-ORF-F2	AACTGCAGGATGACCTCGCGCAATATCGTCG	BACTH assay
AMIS54240-ORF-R2	CCGGATCCTCGCAGCGGCGCAGCACGGCCT	BACTH assay
ssdA-PR1	CGGGATCCATGCCGTGATGCTGTCCGGT	BACTH assay
ssdA-PF2	AACTGCAGGGCGATGCTCAGTGACACCA	BACTH assay
AMIS54240-ORF-F4	GGAATTCCATATGACCTCGCGCAATATCGT	Recombinant protein production
AMIS54240-ORF-R3	CCCAAGCTTTCAGCAGCGGCGCAGCACGGCCT	Recombinant protein production
AMIS25220-ivt-F1	GGAATTCACGATCGCGAAACCGGTCATC	*In vitro* transcription
AMIS25220-ivt-R1	CGCAAGCTTGGATCGATCACTTCTCGGAC	*In vitro* transcription
AMIS68780-ivt-F1	GGAATTCCATCTGAGTACGGTCGATC	*In vitro* transcription
AMIS68780-ivt-R1	CGCAAGCTTCACCGGTCGTGGTCATGATG	*In vitro* transcription

[a] The recognition sequences for restriction enzymes are underlined.

<u>Supplementary Note 1</u>

Glutathione, but not mycothiol, seems to function as a reducing agent for the oxidative stress response in *A. missouriensis.* We found that σ^{SsdA} plays a fundamental role in the oxidative stress response in zoospores. Three genes, *AMIS_39380*, *AMIS_47380*, and *AMIS_72560,* which are under the control of σ^{SsdA}, encode glutamate-cysteine ligases that catalyze the rate-limiting step in the biosynthesis of the antioxidant agent glutathione (1). In the genome sequence of *A. missouriensis*, *AMIS_45940* encodes a glutathione synthase that catalyzes a reaction following the reaction catalyzed by glutamate-cysteine ligase in glutathione biosynthesis. Although mycothiols, instead of glutathione, are produced as critical agents for the control of redox balance among members of *Streptomyces* and several other actinomycetes, including *Mycobacterium* species (2), glutathione in addition to mycothiol has been detected in *Rhodococcus* sp. AD45 (3). Considering that no genes for mycothiol production were found in the *A. missouriensis* genome (2, 4), we believe that *A. missouriensis* produces glutathione to cope with the detrimental effects of oxidative stress.

<u>Supplementary Note 2</u>

No homologues of germinant receptors in *B. subtilis* are encoded in the *A. missouriensis* genome. Spores of *B. subtilis* sense germinant molecules via receptor complexes embedded in their inner membrane (5). Although five germinant receptor operons are encoded in *B. subtilis*, no homologue of the receptor components is encoded in *A. missouriensis*, indicating that *A. missouriensis* zoospores do not rely on the signal transduction pathway involving germinant receptors. We suppose that this is because of the survival strategy employed in the life cycle of *A. missouriensis*. Under laboratory conditions, *A. missouriensis* forms dormant sporangia under desiccated culture conditions, where it can survive and remain dormant for at least several months (Fig. S8). Subsequently, dormant sporangiospores rapidly exit dormancy upon exposure to water. In this activation process, no nutrient molecules from the external environment are required because various buffers with neutral pH (approximately 6.5 to 8.0) are sufficient to induce sporangium dehiscence (our unpublished result). After release from

the sporangia, spores start swimming by means of flagella toward favorable niches, where they resume growth. Therefore, the search for molecules that induce outgrowth and subsequent vegetative growth is temporally separate from the activation of dormant sporangiospores in the life cycle of *A. missouriensis*. In this respect, methyl-accepting chemotaxis proteins (MCPs) embedded in the zoospore membrane may play an important role in the later stages of spore revival because they can induce outgrowth via the sensing of nutrients as chemoattractants (6, 7).

Supplementary References

1. Lu SC. 2013. Glutathione synthesis. Biochim. Biophys. Acta. **1830**:3143-3153.
2. Jothivasan VK, Hamilton CJ. 2008. Mycothiol: synthesis, biosynthesis and biological functions of the major low molecular weight thiol in actinomycetes. Nat. Prod. Rep. **25**:1091-1117.
3. Johnson T, Newton GL, Fahey RC, Rawat M. 2009. Unusual production of glutathione in *Actinobacteria*. Arch. Microbiol. **191**:89-93.
4. Yamamura H, Ohnishi Y, Ishikawa J, Ichikawa N, Ikeda H, Sekine M, Harada T, Horinouchi S, Otoguro M, Tamura T, Suzuki K, Hoshino Y, Arisawa A, Nakagawa Y, Fujita N, Hayakawa M. 2012. Complete genome sequence of the motile actinomycete *Actinoplanes missouriensis* 431^T (= NBRC 102363^T). Stand. Genomic Sci. **7**:294-303.
5. Paidhungat M, Setlow P. 2000. Role of ger proteins in nutrient and nonnutrient triggering of spore germination in *Bacillus subtilis*. J. Bacteriol. **182**:2513-2519.
6. Hayakawa M, Tamura T, Nonomura H. 1991. Selective isolation of *Actinoplanes* and *Dactylosporangium* from soil by using γ-collidine as the chemoattractant. J. Ferment. Bioeng. **72**:426-432.
7. Arora DK. 1986. Chemotaxis of *Actinoplanes missouriensis* zoospores to fungal conidia, chlamydospores and sclerotia. Microbiology. **132**:1657-1663.
