# Supplementary material for: A unique sigma/anti-sigma system in the actinomycete Actinoplanes missouriensis
Source: Nat Commun. 2023 Dec 20;14:8483. doi: 10.1038/s41467-023-44291-y (PMC10733313; doi:10.1038/s41467-023-44291-y)
Supplement: Supplementary file 3 — Description of Additional Supplementary Files [file 41467_2023_44291_MOESM3_ESM.pdf]

Description of Additional Supplementary Files:

Supplementary Data 1: PDB files of the structural models shown in the Figs. 3, S7a, S7d, S7g, S7j, and S7m.

Supplementary Data 2: Genes upregulated in the ΔsipA strain (> 2.0-fold, q value < 0.05; 213 genes)

Supplementary Data 3: Genes downregulated in the ΔsipA strain (> 2.0- fold, q vaule < 0.05; 333 genes)
